# Cost-effectiveness of inhaled oxytocin for prevention of postpartum haemorrhage: a modelling study applied to two high burden settings

**DOI:** 10.1186/s12916-020-01658-y

**Published:** 2020-07-28

**Authors:** Natalie Carvalho, Mohammad Enamul Hoque, Victoria L. Oliver, Abbey Byrne, Michelle Kermode, Pete Lambert, Michelle P. McIntosh, Alison Morgan

**Affiliations:** 1 Centre for Health Policy & Global Burden of Disease Group, School of Population and Global Health, The University of Melbourne, Melbourne, VIC 3010 Australia; 2 Agency for Clinical Innovation, NSW Ministry of Health, Sydney, NSW 2067 Australia; 3 Drug Delivery Disposition and Dynamics, Monash Institute of Pharmaceutical Sciences, Monash University, Parkville, VIC 3052 Australia; 4 Nossal Institute for Global Health, Melbourne School of Population and Global Health, University of Melbourne, Melbourne, VIC Australia

**Keywords:** Maternal health, Global health, Sustainable development goals, Heat-stable uterotonics, Health economic analysis, Ethiopia, Bangladesh

## Abstract

**Background:**

Access to oxytocin for prevention of postpartum haemorrhage (PPH) in resource-poor settings is limited by the requirement for a consistent cold chain and for a skilled attendant to administer the injection. To overcome these barriers, heat-stable, non-injectable formulations of oxytocin are under development, including oxytocin for inhalation. This study modelled the cost-effectiveness of an inhaled oxytocin product (IHO) in Bangladesh and Ethiopia.

**Methods:**

A decision analytic model was developed to assess the cost-effectiveness of IHO for the prevention of PPH compared to the standard of care in Bangladesh and Ethiopia. In Bangladesh, introduction of IHO was modelled in all public facilities and home deliveries with or without a skilled attendant. In Ethiopia, IHO was modelled in all public facilities and home deliveries with health extension workers. Costs (costs of introduction, PPH prevention and PPH treatment) and effects (PPH cases averted, deaths averted) were modelled over a 12-month program. Life years gained were modelled over a lifetime horizon (discounted at 3%). Cost of maintaining the cold chain or effects of compromised oxytocin quality (in the absence of a cold chain) were not modelled.

**Results:**

In Bangladesh, IHO was estimated to avert 18,644 cases of PPH, 76 maternal deaths and 1954 maternal life years lost. This also yielded a cost-saving, with the majority of gains occurring among home deliveries where IHO would replace misoprostol. In Ethiopia, IHO averted 3111 PPH cases, 30 maternal deaths and 767 maternal life years lost. The full IHO introduction program bears an incremental cost-effectiveness ratio (ICER) of between 2 and 3 times the per-capita Gross Domestic Product (GDP) ($1880 USD per maternal life year lost) and thus is unlikely to be considered cost-effective in Ethiopia. However, the ICER of routine IHO administration considering recurring cost alone falls under 25% of per-capita GDP ($175 USD per maternal life-year saved).

**Conclusions:**

IHO has the potential to expand access to uterotonics and reduce PPH-associated morbidity and mortality in high burden settings. This can facilitate reduced spending on PPH management, making the product highly cost-effective in settings where coverage of institutional delivery is lagging.

## Introduction

Despite substantial global progress in reducing maternal mortality over the last two decades, more than a quarter of a million women still die each year as a result of pregnancy- or delivery-related complications [1, 2]. Postpartum haemorrhage (PPH), defined as blood loss of 500 ml or more within 24 h of childbirth, is responsible for one fifth of all maternal deaths and is the leading cause of maternal mortality globally [3]. The World Health Organization (WHO) recommends oxytocin (10 IU) delivered intravenously or intramuscularly to prevent PPH [3].

Injectable oxytocin requires cold chain storage to prevent degradation and to be administered parenterally: two considerations that limit effective utilisation in many low- and middle-income countries (LMICs). Maintenance of a consistent cold chain in LMICs is challenged by resource and infrastructure constraints, and consequently, the quality of oxytocin in these settings is often below international quality specifications [4]. Further, workforce shortages and policies that prohibit administration of injections by some cadres of peripheral health workers limit access to oxytocin for many women in LMICs [5–7]. In settings where skilled health personnel are not available to administer injectable uterotonics, WHO recommends the oral or sublingual administration of misoprostol by community health workers [3]. However, misoprostol is less effective than oxytocin in preventing PPH and is associated with a greater number of side effects [8].

Novel formulations of oxytocin are under development, which aim to be heat-stable and delivered without injection, including a sublingual tablet [9] and dry-powder inhalers [10, 11]. Subject to clinical testing, these products have the potential to deliver clinical protection against PPH without reductions in efficacy that result from inconsistencies in cold chain supply and storage. As non-injectable options for oxytocin administration, these products may facilitate task-shifting to lower tier health workers who may not be able or authorised to deliver an injection. In settings with enabling policy environments and supportive health system structures, these products may be suitable for self-administration through an advanced community distribution model, which has been explored with misoprostol in several countries [12].

Evidence on the expected health gains, costs, and cost-effectiveness of a heat-stable, non-injectable oxytocin product is essential for national-level policy-makers, external donors and development partners working towards reducing maternal mortality. The objective of this study was to estimate the potential impact, costs, and cost-effectiveness of introducing an inhaled oxytocin product (IHO) in two high-burden countries: Bangladesh and Ethiopia. These countries were selected based on several considerations including their high burden of PPH and the accessibility of costing and health data through the networks of the authors. With differing levels of maternal mortality, and unique policy environments leading to important distinctions in delivery location and uterotonic coverage, these countries represent ideal case studies for considering the expected outcomes following implementation of IHO more globally.

## Methods

### Model overview

**Fig. 1:**
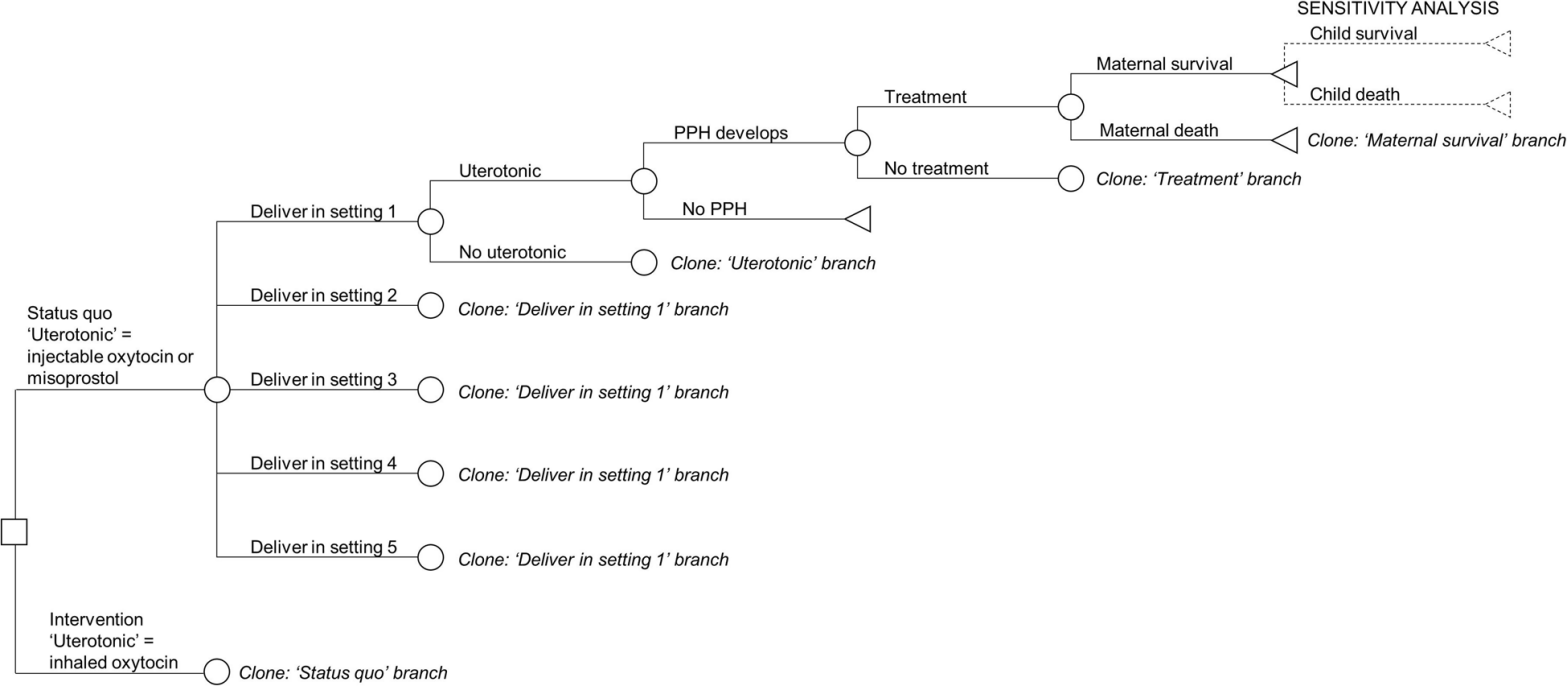
Decision tree for outcomes over a single year, depicting use of inhaled oxytocin versus current uterotonics

A decision analytic model (Fig. 1) was built in Excel (version 16.22) to model the introduction of IHO for the prevention of PPH compared to the status quo in Bangladesh and Ethiopia. For each country, the cohort of women who would experience a birth during a 1-year period is run through the model, going either down the usual care pathway (status quo) or down the intervention pathway (IHO used as uterotonic).

The status quo and intervention scenarios were modelled based on the current service delivery structures and policy contexts in both countries (outlined in Table 1), and take into account the current use of uterotonics within and outside of facilities. To this end, we defined five settings of childbirth and modelled the inhaled oxytocin product to replace the uterotonics used in the status quo (injectable oxytocin or misoprostol) depending on the delivery setting (Table 2). In Bangladesh, introduction of IHO was modelled in all public facilities and at home deliveries with or without a skilled birth attendant (settings 1–4). In Ethiopia, introduction of IHO was modelled in all public facilities and at home deliveries that are attended by HEWs (settings 1–3). These introduction settings for IHO have been designed to align with existing policy in each country relating to the settings where uterotonic use for PPH prevention is authorised.

**Table 1:** Country health system contexts

**Bangladesh**	
Located in South Asia, Bangladesh is the third most populous country in the region and one of the most densely populated countries in the world. A recent national survey reported the maternal mortality ratio in Bangladesh to be 196 deaths per 100,000 live births in 2016, with 31% due to haemorrhage (antepartum and postpartum) [13]. The public service delivery structure includes national, district, upazilla (sub-district), union and ward levels [14]. At the union level, union sub-centres and health and family welfare centres provide the first contact between the population and the health care system and a minority of these facilities offer normal delivery services [15]. At the upazilla level, maternal and child welfare centres and upazilla health complexes typically offer normal delivery services and some are equipped to provide caesarean section. Approximately one third of deliveries occur in a private facility and approximately one half of women give birth outside of a health facility [13]. The Government of Bangladesh has outlined a strategy to scale up misoprostol for the prevention of PPH outside of facilities through an advanced distribution model [16]. A 2015–2016 evaluation showed that community distribution of misoprostol had reached 17% of all births in Bangladesh at this stage of program roll out [13].	
**Ethiopia**	
Ethiopia is located in North-East Africa and, with a population of just over 94 million, it is the second most populous country in Africa. Maternal mortality has decreased substantially in the last decade, and most recent reports estimate a maternal mortality ratio of 422 [17]. The government is the main provider of health care services in the country through a three-tier system consisting of specialist hospitals, general hospitals and primary care units (composed of a network of primary hospitals, health centres and health posts). At the primary care level, emergency obstetric care services are available at some primary hospitals, while health centres provide delivery services and some are equipped to provide basic emergency obstetric care. Each health centre is connected to four health posts, which are staffed by two health extension workers (HEWs). This cadre provide a package of basic curative, promotive and preventative care at the health post or in the home. While national policy in Ethiopia permits use of misoprostol by HEWs, progress towards scale up beyond research areas is uncertain [18, 19]. The Ministry of Health has introduced integrated refresher in-service training to improve the skills of HEWs and to upgrade these health workers from HEW3 to HEW4 (which includes competencies to support skilled attendance at birth). Despite the conduct of pilot programs to explore the feasibility and acceptability of advanced distribution of misoprostol to pregnant women [18, 20], the government of Ethiopia has elected not to adopt this strategy into policy.

**Table 2:** Description of delivery settings that were defined for the purpose of modelling uterotonic coverage in status quo and intervention scenarios. The uterotonics used for prevention of PPH (in non-operative deliveries) at each setting in status quo and intervention scenarios are specified

	Setting 1	Setting 2	Setting 3	Setting 4	Setting 5
	Tertiary-level public facilities	Secondary-level public facilities	Primary health facilities and non-facility births attended by a skilled provider	Non-facility births not attended by a skilled provider	Private sector deliveries
**Settings included in each country**					
Bangladesh	Medical Colleges, Specialised hospitals, District hospitals	Upazilla health complex, Maternal and child welfare centres	Union sub-centres, Rural sub-centres, Union health and family welfare centres, Community clinics, out-of-facility deliveries attended by a medically trained provider	Out-of-facility deliveries attended by a TBA, trained TBA, relative, no-one or other.	Private healthcare facilities
Ethiopia	Specialist hospitals, General hospitals, Primary hospitals	Health centres	Deliveries attended by a HEW (health post or out of facility)	Out-of-facility deliveries attended by a TBA, trained TBA, relative, no-one or other.	Private healthcare facilities
**Uterotonic used for PPH prevention (non-operative deliveries) in status quo**					
Bangladesh	Injectable oxytocin	Injectable oxytocin	Injectable oxytocin or misoprostol	Misoprostol	Injectable oxytocin
Ethiopia	Injectable oxytocin	Injectable oxytocin	Misoprostol	None	Injectable oxytocin
**Uterotonic used for PPH prevention (non-operative deliveries) in intervention scenario**^**a**^					
Bangladesh	Inhaled oxytocin	Inhaled oxytocin	Inhaled oxytocin	Inhaled oxytocin	Injectable oxytocin
Ethiopia	Inhaled oxytocin	Inhaled oxytocin	Inhaled oxytocin	None	Injectable oxytocin

*HEW* health extension worker, *PPH* postpartum haemorrhage, *TBA* traditional birth attendant

^a^In intervention scenario, injectable oxytocin continues to be used for operative deliveries, while inhaled oxytocin is used for non-operative deliveries in settings where roll out is modelled. See appendix (Additional file 1) for estimates of operative delivery rates in each delivery setting

We estimated the costs and cost-effectiveness of IHO from a limited societal perspective, including both health system, household direct medical costs and household non-medical costs related to transport and other delivery-related expenses. Indirect costs due to lost productivity were not modelled. The effectiveness of each uterotonic is calculated based on the coverage at each delivery setting and the efficacy of the drug.

Model outcomes include intermediate clinical events (non-severe PPH, defined as 500–1000-ml blood loss, and severe PPH, defined as blood loss > 1000 ml) and long-term outcomes (PPH-related maternal death, maternal death-related child deaths and life years lost). A 1-year program intervention was modelled, with all costs occurring in the first year, and health benefits modelled over a life-time horizon. Future costs in added life-years were not included, but future health outcomes were discounted at 3% in line with previous economic evaluations in Bangladesh and Ethiopia that have applied a discount rate to benefits [21–23]. The discount rate was varied from 0 to 6% as part of a sensitivity analysis.

Model parameters were informed by the published literature supplemented by in-country interviews to collect local costing data. All costs were adjusted to 2017 US dollars (USD) using country-specific consumer price indices and exchange rates of 80.65 Bangladeshi Taka and 21.73 Ethiopian Birr per USD. Opportunity costs of non-tradable goods taken from other study settings were converted using purchasing power parity. Sensitivity analyses were conducted to determine the robustness of findings to changes in input parameters. In the base case analysis, only health outcomes for the woman were considered, with long-term outcomes for the child modelled in sensitivity analyses.

Incremental cost-effectiveness ratios (ICERs) of IHO introduction, compared to the current use of injectable oxytocin or misoprostol, were calculated in terms of cost per case of PPH avoided, cost per maternal death averted and cost per life year saved. We did not have a reliable way to estimate disability-adjusted life years averted given that the duration of disability (the leading associated disability being anaemia) following a PPH is not well known, and anaemia is often a common pre-existing condition, particularly in Bangladesh [24]. Gross domestic product (GDP) per capita has historically been used as a threshold for determining the cost-effectiveness of an intervention in LMICs, with an intervention categorised as cost-effective if the ICER (expressed as $ per life year saved, or health-adjusted life year saved) falls below 3× GDP/capita and very cost-effective if the ICER falls below 1× GDP/capita [25]. Recent guidance, however, indicates these thresholds are likely to be too high, and much lower thresholds may be more in line with real willingness-to-pay, particularly in LMICs [26]. Recently estimated thresholds for Bangladesh and Ethiopia range from 2 to 50% of GDP/capita [26]. As such, ICERs were compared to several thresholds: 0.25×, 0.5×, and 1× GDP/capita, using Bangladesh’s GDP/capita of US$1516 (2017) and Ethiopia’s GDP/capita of US$768 (2017).

We followed the Consolidated Health Economic Evaluation Reporting Standards guidelines in reporting our findings [27].

All parameters are detailed in the following sections, tables and the appendix (Additional file 1) [13, 16, 17, 28–33], and the model is available to researchers upon request.

### Input parameters

#### Demography, maternal mortality and incidence of PPH

The expected number of births among women of reproductive age (15–49 years) by 5-year age group was calculated based on country-specific demographic estimates and country- and age-specific fertility rates (Table 3). The number of maternal deaths and deaths due to PPH were calculated for each 5-year age group based on country- and age-specific maternal mortality ratios and estimated proportions of maternal deaths due to PPH from country and regional data (Table 3). Deaths unrelated to PPH were assumed to be similar across the status quo and intervention scenarios and were not captured in the model. Number of maternal life years lost was estimated based on the age-specific life expectancy, which was drawn from the WHO Global Health Observatory [39]. A 3% discount rate was applied to number of life years lost, and this discount rate was varied in a sensitivity analysis. Rates of non-severe (500–1000-ml blood loss) and severe PPH (blood loss greater than 1000 ml) were calculated for each delivery setting based on uterotonic efficacy and coverage.

**Table 3:** Input parameters used to model health outcomes

	Bangladesh		Ethiopia	
	Value	Source	Value	Source
**Number of women of reproductive age (15–49) (‘000)**	44,998^a^	UN data 2017 [34]	24,150^a^	UN data 2017 [34]
**Fertility rate of women of reproductive age (15–49)**	73^a^	DHS 2015 [35]	141^a^	DHS 2016 [36]
**Maternal mortality ratio**^**b**^	205^a^	BMMS 2016 [13]	412	EmONC assessment 2016 [17]
**Maternal deaths due to PPH**	27%^a^	BMMS 2016 [13]	31%	EmONC assessment 2016 [17]
**Child survival rate (to age of 12 months)**				
If mother survives	92.4%	Ronsmans et al. 2010 [37]	95.6%	Moucheraud et al. 2015 [38]
If mother dies within 42 days of childbirth	29.6%	Ronsmans et al. 2010 [37]	18.75%	Moucheraud et al. 2015 [38]
**Incidence of PPH without preventative uterotonics**				
Mild	11.3%	Gallos et al. 2018 [8]	11.3%	Gallos et al. 2018 [8]
Severe	5.9%	Gallos et al. 2018 [8]	5.9%	Gallos et al. 2018 [8]
**Risk ratio of mild PPH with uterotonics for prevention**				
Injectable oxytocin	0.61	Gallos et al. 2018 [8]	0.61	Gallos et al. 2018 [8]
Misoprostol	0.75	Gallos et al. 2018 [8]	0.75	Gallos et al. 2018 [8]
Inhaled oxytocin	0.61	Assumption	0.61	Assumption
**Risk ratio of severe PPH with uterotonics for prevention**				
Injectable oxytocin	0.61	Gallos et al. 2018 [8]	0.61	Gallos et al. 2018 [8]
Misoprostol	0.73	Gallos et al. 2018 [8]	0.73	Gallos et al. 2018 [8]
Inhaled oxytocin	0.61	Assumption	0.61	Assumption

*UN* United Nations, *DHS* Demographic and Health Survey, *BMMS* Bangladesh Maternal Mortality Survey, *EmONC* Emergency Obstetric and Neonatal Care, *PPH* postpartum haemorrhage

^a^Age-specific values used (5-year brackets)

^b^Deaths per 100,000 live births

#### Efficacy

The efficacy of injectable oxytocin versus no uterotonic was drawn from a recent Cochrane review (Table 3) [8]. This does not adjust for the possibility that degradation due to inadequate cold storage may result in reduced efficacy from these levels. A 2016 systematic review found an average of 22.3% and 57.5% of oxytocin ampoules collected from Asia and Africa respectively contained below international quality specifications [4]. However, the relationship between oxytocin degradation and clinical effect is not well understood. Thus, it is impossible to credibly model a deviation in efficacy for the injectable oxytocin product. This represents a limitation of the study as the model may underestimate the cost-effectiveness of IHO. The efficacy of misoprostol compared to no uterotonic was drawn from the same Cochrane review. We modelled inhaled oxytocin to have the same efficacy as injectable oxytocin in line with available clinical data demonstrating the similarity in pharmacokinetic profiles for these two administration routes [11].

#### Place of delivery

The percentage of women giving birth in each delivery setting was drawn from national surveys (Table 4). In Ethiopia, there has been a substantial increase in facility-based births, with the most recent health facility survey data from 2016 indicating that most deliveries (66%) occur in health facilities [17], compared to findings from the most recent demographic and health survey (DHS), which indicated that only 26% of births in the preceding 5 years were in facility settings [36]. To allow for extrapolation of these increasing rates of facility deliveries, we modelled 70% of deliveries to occur in a health facility. We modelled the remaining 30% of deliveries to be divided between those that are attended by a HEW (4.5%) and those not attended by a formal provider (25.5%) based on studies conducted in Amhara and Oromia [18]. This represents a small increase in the proportion of deliveries attended by HEW when compared to 2016 DHS data (1.8%), which may be a reasonable assumption based on recent programs to upgrade HEW to the level of skilled birth attendant. Sensitivity analyses were conducted to model realistic lower and upper-bound estimates of deliveries in facilities (40 to 60% in Bangladesh and 60 to 80% in Ethiopia), with the same proportions of deliveries across each out-of-facility and in-facility setting.

**Table 4:** Distribution of births across delivery settings and coverage of uterotonics at each delivery setting in status quo and intervention scenarios. Values modelled in sensitivity analysis are indicated in parentheses

	Setting 1		Setting 2		Setting 3		Setting 4		Setting 5	
	Value	Source	Value	Source	Value	Source	Value	Source	Value	Source
**Bangladesh**										
**Births taking place at setting**	4.0%	BMMS 2016 [13]	9.3%	BMMS 2016 [13]	3.7%	BMMS 2016 [13]	50.2%	BMMS 2016 [13]	32.8%	BMMS 2016 [13]
**Births receiving each uterotonic in status quo**										
Injectable oxytocin	90.2%	Health facility survey [15]	83.9%	Health facility survey [15]	11%	Health facility survey [15]	0%	Assumption	86%	Health facility survey [15]
Misoprostol	0%	Assumption	0%	Assumption	69%	Health facility survey [15] Nasreen et al. 2011 [40]	42%^b^	Quaiyum et al. 2014 [41]	0%	Assumption
**Births receiving each uterotonic in intervention scenario**^**a**^										
Injectable oxytocin	63%	BMMS 2016 [13]	20%	BMMS 2016 [13]	0%	Assumption	0%	Assumption	86%	BMMS 2016 [13]
Misoprostol	0%	Assumption	0%	Assumption	0%	Assumption	0%	Assumption	0%	Assumption
Inhaled oxytocin	28%	Assumption	64%	Assumption	80%	Assumption	42% ^b^	Assumption	0%	Assumption
**Ethiopia**										
**Births taking place at setting**	9.5%	EmONC assessment 2016 [17]	58.4%	EmONC assessment 2016 [17]	4.5%	Sibley et al. 2014 [18]	25.5%	Sibley et al. 2014 [18]	2.1%	EmONC assessment 2016 [17]
**Births receiving each uterotonic in status quo**										
Injectable oxytocin	81%	EmONC assessment 2016 [17]	81%	EmONC assessment 2016 [17]	0%	Assumption	0%	Assumption	81%	EmONC assessment 2016 [17]
Misoprostol	0%	Assumption	0%	Assumption	84%^c^	Health facility survey [42]	0%	Assumption	0%	Assumption
**Births receiving each uterotonic in intervention scenario**^**a**^										
Injectable oxytocin	2%	EmONC assessment 2016 [17]	0%	Assumption	0%	Assumption	0%	Assumption	81%	EmONC assessment 2016 [17]
Misoprostol	0%	Assumption	0%	Assumption	0%	Assumption	0%	Assumption	0%	Assumption
Inhaled oxytocin	78%	Assumption	81%	Assumption	84% ^c^	Assumption	0%	Assumption	0%	Assumption

*BMMS* Bangladesh Maternal Mortality Survey, *EmONC* Emergency Obstetric and Neonatal Care

^a^Coverage of uterotonics have been modelled on the basis of injectable oxytocin being used for operative deliveries, while inhaled oxytocin is used for non-operative deliveries in settings where roll-out is modelled. See appendix (Additional file 1) for estimates of operative delivery rates in each delivery setting

^b^Varied in sensitivity analyses from 21 to 63%; (uniform distribution for probabilistic sensitivity analysis)

^c^Varied in sensitivity analyses from 75 to 95%; (uniform distribution for probabilistic sensitivity analysis)

#### Current use of uterotonics

Current use (coverage) of injectable oxytocin and misoprostol at each level of facility was drawn from health facility reviews (Table 4) [15, 17, 42]. For out-of-facility births, we assume only misoprostol is available for a subset of deliveries. In Bangladesh, current estimates of misoprostol coverage at out-of-facility deliveries reflect an incomplete stage of program roll out. To estimate the coverage that may be achieved at ‘full scale’, we modelled misoprostol use at 50% of the coverage level found in intervention studies in rural Bangladesh (83.7%), varying this scaling factor from 25 to 75% in sensitivity analyses [41]. In Ethiopia, we assume 84% of women with HEW-assisted deliveries receive misoprostol, based on health facility survey data of the availability of various reproductive health medicines at health posts [42]. This was varied in a sensitivity analysis to account for the uncertainty surrounding this assumption. We assume that women who delivered themselves or with another type of attendant do not receive misoprostol.

#### Rollout of IHO

For the intervention scenarios, we modelled a direct substitution of current uterotonics with IHO for non-operative deliveries, assuming that coverage of IHO would be equivalent to current uterotonics. We assume IHO would not be used for caesarean deliveries, and thus, there is no effect of the intervention on caesarean section deliveries.

#### Cost

Three categories of costs were included in the analysis: intervention up-front costs (advocacy and training), ongoing PPH prevention costs (commodity costs, wastage and disposal), and PPH treatment costs (Table 5). Further detail is available in the appendix (Additional file 1).

**Table 5:** Input parameters used to model costs. Up-front costs were modelled for the inhaled oxytocin product only. All costs are in 2017 USD

	Bangladesh		Ethiopia	
	Value	Source	Value	Source
**Intervention up-front costs**				
**Advocacy costs**^**a**^	$ 321,105	MoH informant	$ 80,563	MoH informant
**Up-front training costs**^**a**^	$ 96,255	MoH informant and health sector plan [16]	$ 39,531	MoH informant
**Health worker training costs (per facility)**^**a**^	$ 226	Health sector plan [16]	$ 324	MoH informant
**Number of facilities providing delivery care for each delivery setting**				
**Setting 1**	78	Health Bulletin [28], Health facility survey [15]	95	EmONC assessment 2016 [17]
**Setting 2**	471	Health Bulletin [28], Health facility survey [15]	3567	EmONC assessment 2016 [17]
**Setting 3**	1828	Health Bulletin [28], Health facility survey [15]	N/A^b^	
**Ongoing PPH prevention costs**				
**Drug cost (per dose)**				
**Injectable oxytocin**	$ 0.34^c^	Drug administration informant	$ 0.37^d^	Public supply agency informant
**Misoprostol**	$ 0.34^e^	Drug administration informant	$ 0.60^f^	International drug price indicator
**Inhaled oxytocin**	$ 0.50^g^	Assumption	$ 0.50^g^	Assumption
**Disposal costs (per 100 doses)**				
**Injectable oxytocin**	$ 1.33	Sarker et al. 2015 [43]	$ 0.28	Sarker et al. 2015 [43]
**Misoprostol**	$ -	Assumption	$ -	Assumption
**Inhaled oxytocin**	$ 1.42	Sarker et al. 2015 [43]	$ 0.42	Sarker et al. 2015 [43]
**Wastage rates**				
**Injectable oxytocin**	5%	Pecenka et al. 2017 [44]	5%	Pecenka et al. 2017 [44]
**Misoprostol**	5%	Vlassoff et al. 2016 [45]	5%	Vlassoff et al. 2016 [45]
**Inhaled oxytocin**	7%	Local clinicians	7%	Local clinicians
**PPH treatment costs**				
**% of PPH cases after a facility birth that receive treatment**^**h**^	90%	Assumption	90%	Assumption
**% of PPH cases after a home birth that seek treatment in a facility**^**h**^				
**Public**	32.8%	BMMS 2016 [13]	50.5%	Worku et al. 2013 [46]
**Private**	44.1%	BMMS 2016 [13]	1.6%	Worku et al. 2013 [46]
**Average length of hospital stay**				
**Mild**	2 days	Hospital administrators	2 days	Hospital administrators
**Severe**	5 days	Hospital administrators	5 days	Hospital administrators
**Cost of treating mild PPH**^**a**^				
**Public**	$79	Hospital administrators and clinicians	$31	Akalu et al. 2012 [31] Pearson et al. 2011 [32] Lara et al. 2007 [33]
**Private**	$122	Hospital administrators and clinicians	$73	Akalu et al. 2012 [31] Pearson et al. 2011 [32] Lara et al. 2007 [33]
**Cost of treating severe PPH**^**a**^				
**Public**	$176	Hospital administrators and clinicians	$199	Akalu et al. 2012 [31] Pearson et al. 2011 [32] Lara et al. 2007 [33]
**Private**	$272	Hospital administrators and clinicians	$356	Akalu et al. 2012 [31] Pearson et al. 2011 [32] Lara et al. 2007 [33]

*MoH* Ministry of Health, *EmONC* Emergency Obstetric and Neonatal Care, *PPH* postpartum haemorrhage, *BMMS* Bangladesh Maternal Mortality Survey

^a^See appendix (Additional file 1) for more detail. Varied by ± 25% of base case in sensitivity analysis (uniform distribution of probabilistic sensitivity analysis)

^b^Assume HEW attend trainings at health centres rather than health posts

^c^Two × 5 IU ampoules (0.14 USD each) plus one syringe and needle (0.07 USD)

^d^One × 10 IU ampoule (0.34 USD each) plus syringe and needle (0.03 USD)

^e^Two × 200 μg tablets at 0.17 USD each

^f^Three × 200 μg at 0.20 USD per tablet

^g^Varied in a sensitivity analysis from 0.25 to 1.00 USD (uniform distribution for probabilistic sensitivity analysis)

^h^Varied the total care-seeking by ± 10 percentage points of base case in sensitivity analysis (uniform distribution for probabilistic sensitivity analysis). Distribution across private/public facilities maintained at same proportion as base case

We considered the cost of advocacy required for sensitisation of key stakeholders at all levels (national policy makers, sub-national program managers and community members) through workshops and meetings. Training costs included the costs of preparative activities (curriculum development and training of trainers) in addition to the in-service training of healthcare providers (upfront and refresher trainings). In-service training costs were applied to the number of facilities where introduction of the drug was modelled. Training costs were drawn from a combination of literature sources and insights from experts in each country.

Commodity costs include the uterotonic (injectable oxytocin, misoprostol and IHO) and the syringe and needle needed for administration of injectable oxytocin. Dosage of each drug administered was modelled to be consistent with standard guidelines of practice in each country. Public and private sector prices were included and applied to the appropriate delivery setting. The final price of IHO is yet to be determined. In the base case analysis, we model the price of IHO at US$ 0.50. A sensitivity analysis was conducted to model additional price points of $0.25 and $1.

Disposal costs were based on required method of disposal: syringes to be incinerated and inhalers to be disposed of without incineration. The cost of waste disposal for injectable and inhaled oxytocin was estimated from the disposal costs of a vaccination program in Bangladesh [43], which reports on incineration and non-incineration costs associated with disposal of cholera vaccine vials. The cost of disposal associated with misoprostol was considered negligible.

Wastage costs were included to account for drugs compromised by heat, expiry date, breakage and wear and tear. Wastage rates for injectable oxytocin and misoprostol were modelled at 5% based on studies of medical ampoule wastage rates [44]. For inhaled oxytocin, a higher wastage rate (7%) was assumed based on expert opinion that the inhaler may be discarded at a slightly higher rate than the oxytocin ampoule due incorrect use of the device (Table 5).

Finally, we included the location-specific cost of PPH treatment. We used a micro-costing approach to model the costs of treating mild or severe PPH in a public or a private facility drawn from a combination of literature sources and in-country consultations with relevant informants. Interviews were conducted in September 2017 (Bangladesh) and November 2017 (Ethiopia) with senior Ministry of Health personnel, hospital administrators and clinicians providing obstetric care at private and public hospitals. Costs modelled include direct medical and non-medical expenditures (e.g. food for the mother and accompanying relatives, transport, tips, accommodation for accompanying relatives) [31–33]. We did not consider indirect costs associated with lost productivity. Treatment costs were varied in sensitivity analyses.

To determine the number of women treated, we assumed 90% of women experiencing PPH after giving birth in a facility will receive treatment there. We drew from the literature and country surveys to estimate the number of women seeking care after a home birth in each country. All care-seeking estimates were varied in sensitivity analyses.

### Sensitivity analyses

Several sensitivity analyses were carried out to account for the uncertainty in the model input parameters and to test the impact of different scenarios or structural aspects of the model on outcomes.

#### Deterministic sensitivity analyses

The final cost of IHO is currently unknown, so this parameter was varied from $0.25 to $1.00 in both countries. A threshold analysis was conducted to determine the price threshold for cost-effectiveness of the product. Given up-front intervention costs were found to vary substantially across both countries, we modelled an uncertainty range of ± 25% of all program introduction costs, including both advocacy and training costs. The proportion of women seeking care following a PPH was varied by ± 10 percentage points for all births in and out of a facility. Treatment costs for non-severe and severe PPH in a health facility were varied by ± 25% of base case estimates.

#### Scenario analyses

We varied the coverage of misoprostol (in status quo) and IHO (in intervention scenario) for home births with an unskilled attendant in Bangladesh and with HEW in Ethiopia. For Bangladesh, we modelled a range from 25 to 75% of the misoprostol coverage level found in intervention studies [38]. For Ethiopia, we model coverage of uterotonics with HEW to range from 75 to 95%. As previously explained, we also varied the proportion of deliveries in facilities in both countries.

#### Structural uncertainty analyses

We present results with and without child health outcomes included. The number of child deaths was estimated based on country-specific longitudinal studies which report on the association between maternal death and child survival (Table 3). The number of child life years lost was estimated based on country-specific life expectancy at birth [47]. A 3% discount rate was applied to number of life years lost, which was varied, along with the 3% discount rate applied to maternal life years lost, from 0 to 6% as part of a second structural sensitivity analysis.

We also present results with and without the up-front introduction costs (advocacy and training), to provide an indication of the cost-effectiveness of IHO after several years, when training of health workers is included within the routine curricula and only recurrent costs become relevant.

#### Probabilistic sensitivity analysis

Finally, we conducted a probabilistic sensitivity analysis to evaluate the joint uncertainty of input parameters simultaneously. We ran 10,000 simulations, varying all key parameters described above in the deterministic sensitivity analyses as well as those varied in the scenario analyses, using uniform distributions for all parameters. Probabilistic sensitivity analyses were run separately for each of the structural analyses specified above apart from discount rate, which was kept at 3% across all probabilistic sensitivity analyses. Results were presented as cost-effectiveness acceptability curves.

## Results

In the base case analysis, IHO introduction would avert over 18,500 PPH cases in Bangladesh annually, while just over 3000 PPH cases would be avoided in Ethiopia (Table 6). Our model predicts an estimated 76 PPH-related maternal deaths would be averted in Bangladesh annually, and 30 deaths averted in Ethiopia each year, a reduction of 4.2% and 0.7% of the current number of PPH-related deaths, respectively. These results translate into nearly 2000 maternal life years saved over the women’s remaining lifetime from a 1-year IHO program in Bangladesh and 767 maternal life years saved in Ethiopia (Table 6).

**Table 6:** Estimated maternal and child health benefits of a 1-year inhaled oxytocin introduction program over a lifetime horizon with a 3% discount rate

	Bangladesh			Ethiopia		
	Status quo	Intervention	Averted	Status quo	Intervention	Averted
**PPH cases, non-severe**	291,978	278,813	**13,165**	297,868	295,672	**2197**
**PPH cases, severe**	150,947	145,467	**5479**	155,178	154,264	**914**
**Maternal deaths**	1806	1730	**76**	4418	4388	**30**
**Maternal life years lost**	46,429	44,475	**1954**	111,767	111,000	**767**
**Child deaths**	1135	1088	**48**	3394	3371	**23**
**Child life years lost**	33,480	32,071	**1409**	97,284	96,616	**668**

*PPH* postpartum haemorrhage

In terms of costs, the current spending on uterotonics for PPH prevention and the costs of introducing an IHO program are much lower than the estimated costs of PPH treatment (Table 7). In Bangladesh, the cost savings from reduced spending on treatment due to IHO introduction exceed the additional cost of IHO introduction. As a result, IHO was found to be cost-saving compared to the status quo in Bangladesh, when replacing the current use of uterotonics in public facilities and home births with and without skilled attendants. A threshold analysis indicated that IHO ceases to be a cost-saving intervention when the price of the product exceeds $1.15 (USD); however, it remains highly cost-effective up to a price of $1.90 (at which point the ICER = 25% of the GDP per capita).

**Table 7:** Estimated costs and cost-effectiveness ratios of inhaled oxytocin introduction in each setting from a societal perspective and over a lifetime horizon, 3% discount rate

	Bangladesh			Ethiopia		
	Status quo	Intervention	Incremental	Status quo	Intervention	Incremental
**Costs, $000**						
**Intervention start-up costs**^**a**^		955	955		1308	1308
**On-going PPH prevention**^**b**^	783	985	202	840	1108	268
**PPH treatment**	52,479	50,606	− 1873	31,141	31,007	− 134
**Total costs**	53,262	52,546	− 716	31,981	33,423	1443
**ICERs**						
**$ per PPH case averted**		Cost-saving			464	
**$ per maternal death averted**		Cost-saving			47,557	
**$ per maternal life year saved**		Cost-saving			1880	
**$ per maternal and child life year saved**		Cost-saving			1005	
**ICERs (ongoing costs only)**						
**$ per PPH case averted**		Cost-saving			43	
**$ per maternal death averted**		Cost-saving			4435	
**$ per maternal life year saved**		Cost-saving			175	
**$ per maternal and child life year saved**		Cost-saving			94	

Societal costs include public and private sector costs, and household medical and direct non-medical out of pocket costs in public and private sectors

*ICER* incremental cost-effectiveness ratio, *PPH* postpartum haemorrhage

^a^Training and advocacy costs

^b^Commodity (uterotonics), disposal and wastage costs

In Ethiopia, IHO introduction had an ICER between two and three times the GDP per capita, indicating it is unlikely to be a cost-effective strategy. A price of $0.08 would be required for IHO to be considered a cost-effective intervention in Ethiopia based on a 1 × GDP per capita threshold. Excluding up-front introduction costs related to advocacy and training, IHO had an ICER of under 25% per capita GDP in Ethiopia, indicating a recurring program is likely to become cost-effective after several years of implementation, once IHO has become well-integrated into the health personnel training curricula.

### Sensitivity analysis

**Fig. 2:**
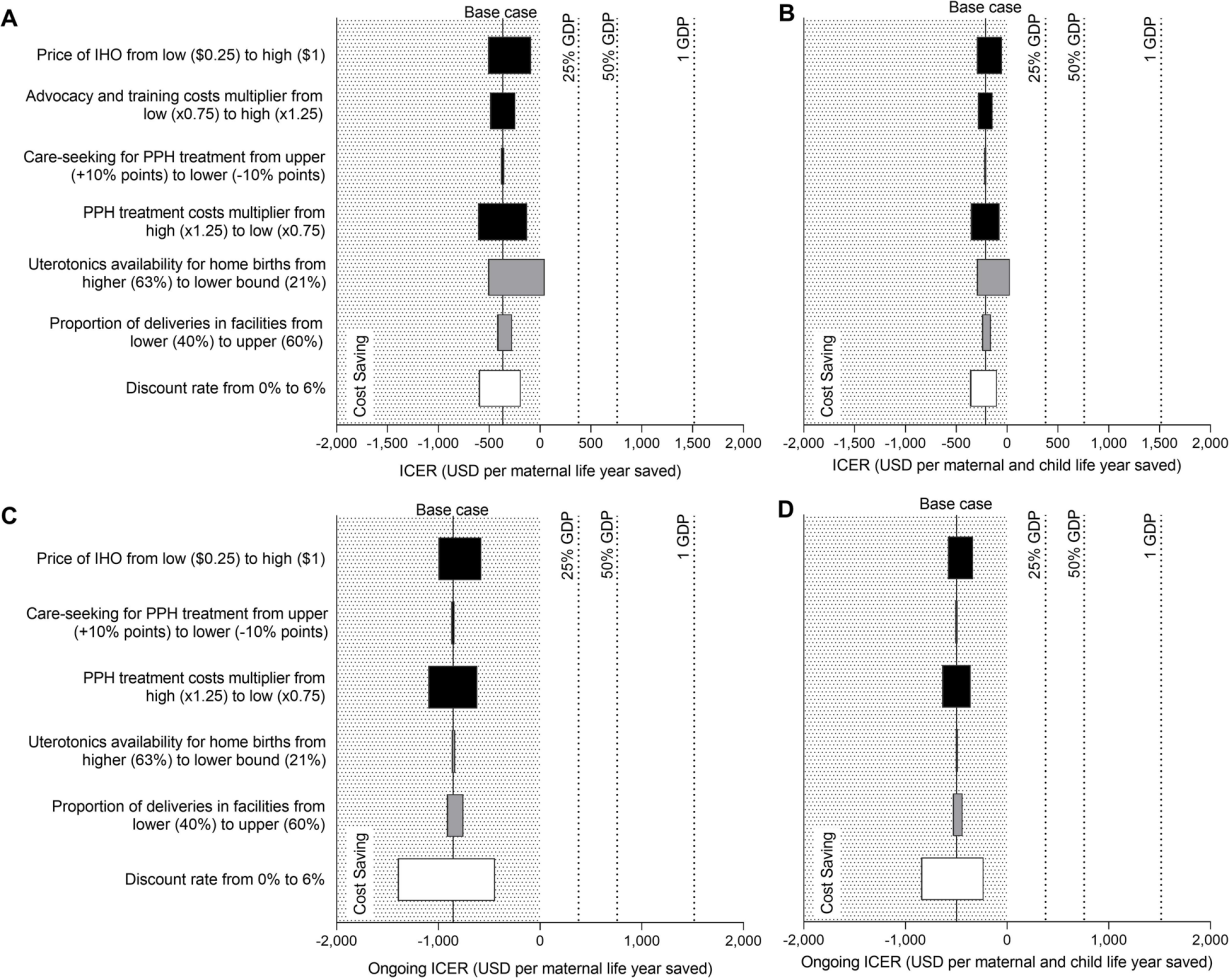
One-way sensitivity analysis of key input parameters on incremental cost-effectiveness ratio of inhaled oxytocin introduction in Bangladesh. **a** Incremental cost per maternal life year saved. **b** Incremental cost per maternal and child life year saved. **c** Incremental cost per maternal life year saved when including only ongoing costs. **d** Incremental cost per maternal and child life year saved when including only ongoing costs. Black bars represent deterministic sensitivity analyses; grey bars represent scenario analyses; white bars represent structural uncertainty analyses. Solid vertical line represents base case; dashed vertical lines represent cost-effectiveness thresholds of 25%, 50% and 1 GDP per capita. GDP, gross domestic product; ICER, incremental cost-effectiveness ratio; IHO, inhaled oxytocin; PPH, postpartum haemorrhage

**Fig. 3:**
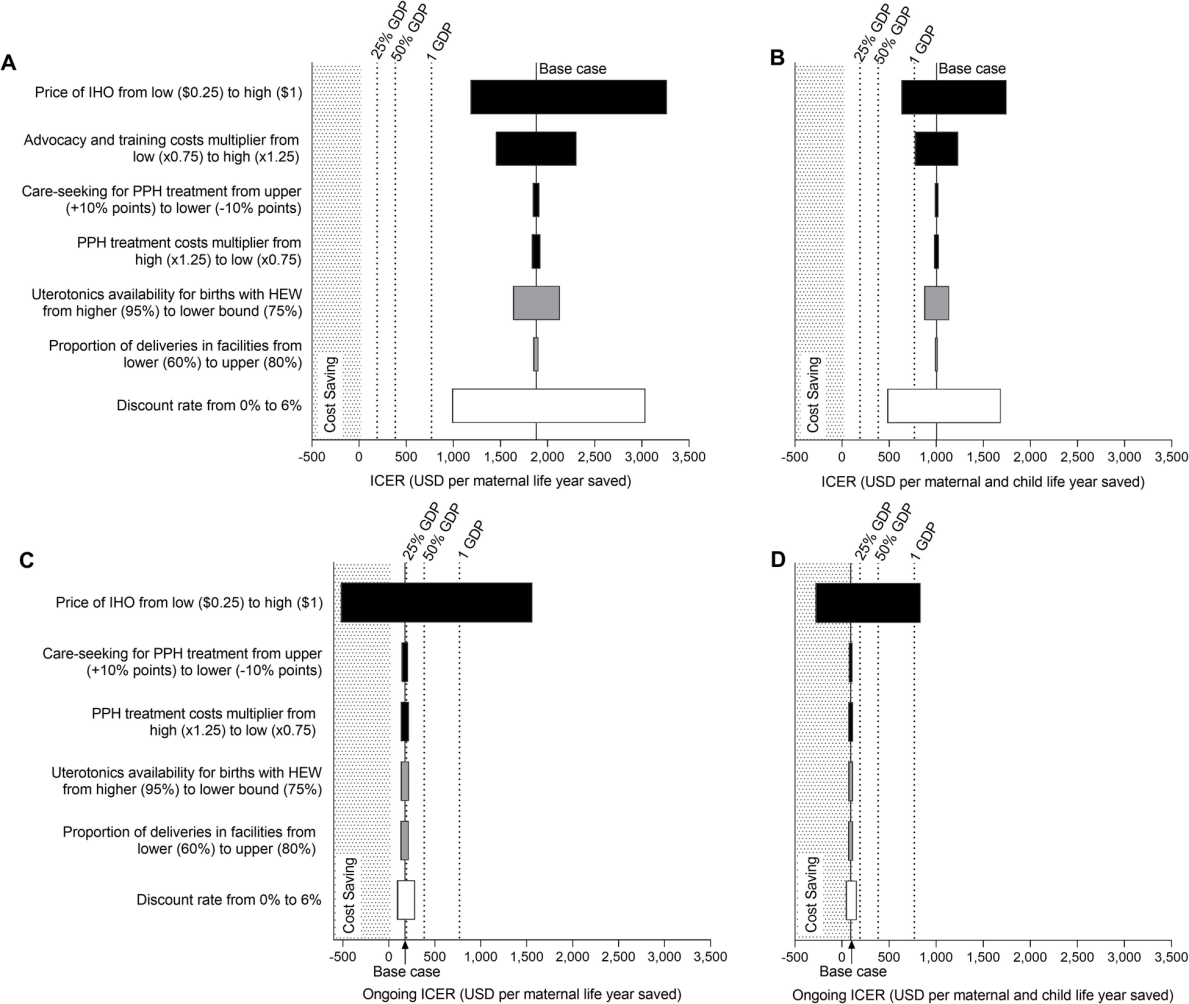
One-way sensitivity analysis of key input parameters on incremental cost-effectiveness ratio of inhaled oxytocin introduction in Ethiopia. **a** Incremental cost per maternal life year saved. **b** Incremental cost per maternal and child life year saved. **c** Incremental cost per maternal life year saved when including only ongoing costs. **d** Incremental cost per maternal and child life year saved when including only ongoing costs. Black bars represent deterministic sensitivity analyses; grey bars represent scenario analyses; white bars represent structural uncertainty analyses. Solid vertical line represents base case; dashed vertical lines represent cost-effectiveness thresholds of 25%, 50% and 1 GDP per capita. GDP, gross domestic product; HEW, health extension workers; ICER, incremental cost-effectiveness ratio; IHO, inhaled oxytocin; PPH, postpartum haemorrhage

See Figs. 2 and 3 for the one-way sensitivity analyses results for Bangladesh and Ethiopia, respectively, with results presented both with and without child health outcomes included. IHO introduction remained cost-saving in Bangladesh across all sensitivity analyses conducted, with one exception: assuming less use of uterotonics (misoprostol in the status quo, IHO in the intervention scenario) at home births without a trained provider present. At the lower bound value modelled for this parameter (21% coverage), the ICER rose to $47 per maternal life year saved, still highly cost-effective at 3% of GDP/capita.

**Fig. 4:**
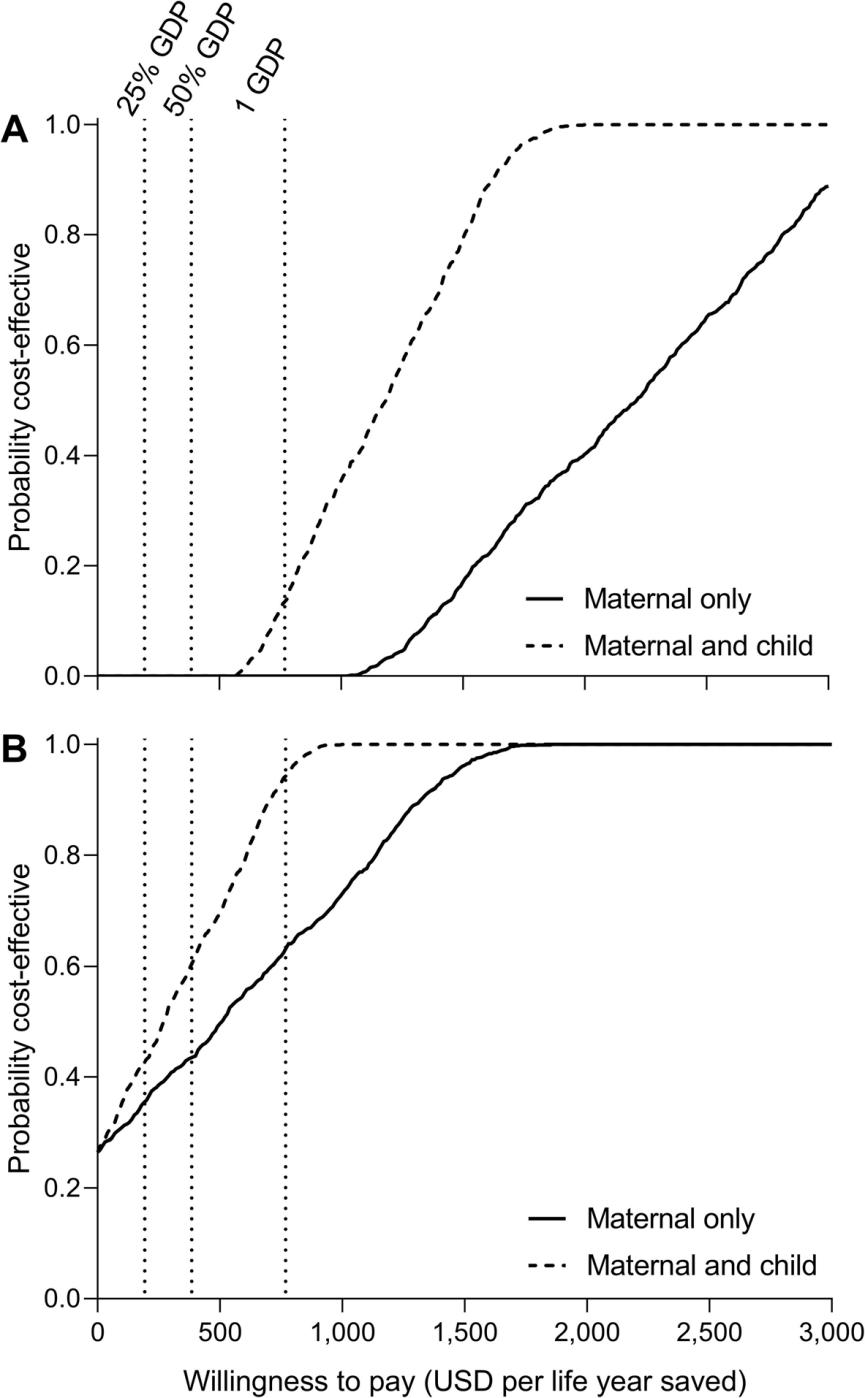
Cost-effectiveness acceptability frontier showing the probability that inhaled oxytocin is cost-effective for different willingness to pay thresholds in Ethiopia. Willingness to pay is shown in terms of USD per maternal life years saved (solid line) or maternal and child life years saved (dashed line). **a** The base case analysis, where all implementation costs are included. **b** Only ongoing costs are included in the analysis. A 3% discount rate was used. Dashed vertical lines represent 25%, 50% and 1 GDP per capita. GDP, gross domestic product

In Ethiopia, the most favourable results were obtained at 0% discount rate (ICER of $990 per maternal life year saved) or at a price of $0.25 for IHO (ICER of $1187 per maternal life year saved). In both cases, with the ICER still above 1.3 times the per capita GDP in Ethiopia, IHO is still unlikely to be cost-effective based on a per-capita GDP threshold. Findings were most sensitive to the price of IHO and the choice of discount rate applied to the future live years saved from a maternal (and child) death. The ICER of IHO introduction in Ethiopia was only found to have an ICER of less than one GPD per capita under a subset of “combined” favourable scenarios, such as when including child health gains in addition to modelling a lower cost of IHO at $0.25, or when including child health gains and modelling a 0% discount rate. From an on-going program perspective, IHO would likely be cost-effective when the advocacy and training introduction costs were excluded and even cost-saving when these program introduction costs were excluded and IHO was modelled at $0.25 per dose. Figure 4 shows the probability of IHO being cost-effective in Ethiopia across a range of willingness-to-pay thresholds tied to Ethiopia’s GDP per capita. Findings from the probabilistic sensitivity analysis (Fig. 4) show that considering maternal outcomes only, IHO has only a 10% probability of cost-effectiveness in Ethiopia at a willingness to pay threshold of twice GDP per capita. Considering both maternal and child health outcomes, IHO has less than a 10% probability of being cost-effective at a willingness to pay threshold of GDP per capita. From an on-going program perspective, IHO is over 60% likely to be cost-effective based on a one times GDP per capita threshold.

## Discussion

This is the first study to comprehensively model the cost-effectiveness of an inhaled oxytocin product for the prevention of PPH in Bangladesh and Ethiopia. In both countries, introduction of inhaled oxytocin to replace injectable oxytocin and misoprostol for PPH prevention was predicted to result in a modest decrease in the maternal deaths and a reduction in the number of PPH cases. No health outcome gains were associated with replacement of injectable oxytocin with the inhaled product at higher level health facilities, as these two oxytocin formulations were modelled to have equivalent efficacy. However, this does not take into consideration the likelihood that compromised cold temperature maintenance during transport and storage may have led to degradation of injectable oxytocin. Several studies have indicated that the quality of oxytocin product available in LMICs is compromised—in part due to inadequate cold chain storage [4, 48, 49]. A 2016 systematic review found an average of 22.3% and 57.5% of oxytocin ampoules collected from Asia and Africa respectively contained below the specified content of oxytocin [4]. However, there are no published studies investigating the impact of poor quality oxytocin on clinical outcomes, and research in this area is challenged by ethical considerations. Thus, while it is not possible to reliably model the health impact associated with replacement of injectable oxytocin with a heat stable product, it can be assumed that health outcomes could be improved to a greater extent that predicted by this model.

Consequently, all health gains were accrued through the replacement of misoprostol with inhaled oxytocin, the latter of which has a greater efficacy for the prevention of PPH. In Bangladesh, this represents a large number of deliveries, encompassing a proportion of home births in addition to deliveries in a subset of primary health facilities that are currently using a mix of oxytocin and misoprostol. In contrast, even small health gains were not seen in facility settings in Ethiopia due to the assumption that misoprostol is not used in lower level facilities. In addition, only a small proportion of deliveries are attended by HEWs (who are assumed to use misoprostol for prevention of PPH), and as such, switching to IHO results in very small health gains overall in Ethiopia as compared to Bangladesh where wider access to IHO was modelled through an advanced community distribution approach.

In Bangladesh, adoption of the inhaled oxytocin product was predicted to be a cost saving, a finding driven by reduced spending on PPH treatment that is made possible by averting cases of PPH. These findings were robust to different sensitivity analyses carried out including those affecting total spending on PPH treatment (per case cost of PPH and percentage of cases seeking treatment).

In Ethiopia, the full program of IHO introduction was predicted to cost greater than double the GDP per capital per maternal life year saved and thus is unlikely to be considered a cost-effective intervention. When including child deaths likely to be averted, IHO introduction was still found to have a cost-effectiveness ratio of greater than Ethiopia’s GDP per capita. Across all sensitivity analyses carried out, the intervention was only found to have an ICER of less than one GDP per capita under a subset of “combined” favourable scenarios (consideration of both maternal and child health outcomes, coupled with a reduced discount rate for future benefits or lower price of IHO). The unfavourable cost-effectiveness ratio for inhaled oxytocin in Ethiopia is largely driven by the high up-front costs associated with product introduction. When considering a scenario several years in the future, when IHO has been fully integrated into the health system, the ICER of the product is less than a quarter of the country’s GDP per capita. Thus on an ongoing basis, the product could be considered a cost-effective intervention for Ethiopia.

The up-front costs of training in Ethiopia were predicted by this model to be almost double those in Bangladesh. The number of facilities where training is required to be administered in Ethiopia exceeds Bangladesh by over 1200, contributing to the overall high costs of health worker training in Ethiopia. The mechanisms employed to administer up-front training for a new product, particularly in vast geographies with a large number of health facilities, should be given careful consideration to maximise the potential for products to deliver cost-effective health impacts. Cost-effective strategies will likely entail ‘bundling’ training for multiple products and services together, such that costs can be diffused across a wider set of health impacts.

There are considerable differences in the demographic and policy contexts between Bangladesh and Ethiopia that allow for translation of results to a variety of other countries. In Bangladesh, rates of out-of-facility deliveries are high (> 50%) and the policy environment will likely support roll out of IHO to these settings. Together, these attributes are a significant driving force for the cost-saving that is predicted, as a large proportion of births gain access to a uterotonic with gold standard efficacy against PPH. Similar cost-effectiveness ratios may be expected in other high-burden countries such as Nigeria, Afghanistan and Pakistan, where rates of out-of-facility deliveries and uterotonic policy contexts are similar to Bangladesh. In contrast, in Ethiopia, relatively few births take place outside of facility settings, and policy support for availability of uterotonics in community settings is limited to births attended by a skilled provider (representing a small portion of births). Thus, the cost-effective ratios predicted for Ethiopia may be similar to those expected in countries such as India, Indonesia, Kenya and the Democratic Republic of Congo.

There are several important strengths and limitations to this modelling exercise that warrant further discussion. Our decision analytic model does not capture other maternal complications including antenatal haemorrhage, or quantify the morbidity impacts of PPH due to anaemia. In addition, we do not model the costs of side effects associated with uterotonics, which have been previously estimated to significantly improve the cost-effectiveness of oxytocin compared to misoprostol due to the side effect profile associated with the latter, which includes shivering, fever, nausea and vomiting [50]. These simplifications are likely to underestimate the true health benefits of IHO and thus represent a conservative simplification.

Cold chain costs were not modelled as storage costs were thought to apply equally to both scenarios given the continued requirement for cold chain systems (for injectable oxytocin required for operative delivery as well as other temperature sensitive drugs) despite introduction of inhaled oxytocin. Further, the lack of reliable costing by volume data for refrigerated and non-refrigerated transport limited credible estimations of cold chain supply costs. Omission of cold chain costs from the model may lead to an underestimation of the cost-effectiveness of the IHO product. However, the decision not to include this cost item is supported by previous studies that have shown that negligible cold chain savings are associated with the replacement of a subset of temperature-sensitive medicines with heat-stable formulations [51, 52].

We model a 1-year program of IHO introduction, applying an assumption that all costs are incurred and maximal coverage is reached during this period. We acknowledge this to be a simplification, as realistically such a program may need greater than 12 months to scale-up and thus costs are likely to be incurred over multiple years, with health gains increasing to reach our estimates over this time. We also show results of an IHO program running at scale and fully integrated into the existing maternal health program, by modelling the costs and cost-effectiveness of the intervention with and without the start-up training costs.

A strength of this analysis is that it gives consideration to both maternal and child health outcomes. A recent systematic review of strategies to improve maternal or newborn health in LMICs shows that most cost-effectiveness analyses report on either maternal or child health outcomes, but rarely both [53]. This approach underestimates of the true magnitude of effect of a maternal health intervention considering that child mortality associated with a maternal death is consistently high across all reported studies [37, 38, 54, 55].

The model assumes that IHO has equivalent efficacy to an injection of oxytocin based on research showing that these two administration routes give rise to comparable systemic concentrations of oxytocin over time in healthy volunteers [11]. The potential for inhaled delivery of peptides to match efficacy profiles of parenteral routes of administration has been demonstrated with inhaled insulin [56]. However, the efficacy of IHO in comparison to an intramuscular injection of oxytocin is yet to be demonstrated and as such the results of this economic analysis may require revision pending the results of large-scale efficacy trials in postpartum women.

The model assumes that there is no difference in the feasibility and acceptability of administering IHO to women immediately after childbirth compared to the uterotonics used in the status quo (injectable oxytocin and sublingual misoprostol). Qualitative research conducted in Myanmar supports this assumption, with healthcare providers and community members suggesting that IHO is likely to be largely acceptable and feasible to use [57]. Yet there are potential implementation challenges of IHO particularly in settings where a skilled birth attendant is managing the labour and delivery without any assistance, and relying on a mother or other family member being able to follow instructions for IHO administration at the time of birth. While this may pose a challenge to effective administration of a full therapeutic dose of IHO, the model includes costs for community sensitisation activities to improve the likelihood of successful administration. Nevertheless, local feasibility and acceptability studies in each context would be important undertakings alongside product introduction.

## Conclusions

In summary, this work provides valuable insights into the potential cost-effectiveness of IHO in different health system contexts. In settings like Bangladesh, where a significant number of women currently lack access to oxytocin for the prevention of PPH at the time of birth, IHO is a cost-saving intervention, as health impacts are accompanied by a substantial reduction in spending on PPH treatment. In the Ethiopian context, the product may not be considered a cost-effective intervention until the product is fully integrated into the health system and up-front introduction costs are no longer incurred. For both country contexts, our findings likely represent an underestimate of the health impacts and cost-effectiveness of IHO, given that it has not been possible to model the impact of inadequate cold storage of injectable oxytocin in our estimate of health outcomes in the status quo. Further research to understand the true cost of poor quality oxytocin would aid in the evaluation of the cost-effectiveness of IHO and other heat stable uterotonics.

## Supplementary information

**Additional file 1: Supplemental appendix.** This appendix provides further detail of model inputs relating to rates of caesarean section delivery, start-up costs for IHO introduction and costs of PPH treatment.

## Data Availability

The model developed and used in this study is available from the corresponding author on reasonable request.

## Abbreviations

DHS: Demographic and health survey
GDP: Gross domestic product
HEW: Health extension worker
ICER: Incremental cost-effectiveness ratio
IHO: Inhaled oxytocin
LMIC: Low- and middle-income countries
MMR: Maternal mortality ratio
PPH: Postpartum haemorrhage
SDG: Sustainable Development Goal
USD: United States dollar
WHO: World Health Organization
