# Supplementary material for: Cost-effectiveness of inhaled oxytocin for prevention of postpartum haemorrhage: a modelling study applied to two high burden settings
Source: BMC Med. 2020 Jul 28;18:201. doi: 10.1186/s12916-020-01658-y (PMC7385867; doi:10.1186/s12916-020-01658-y)
Supplement: Supplementary file 1 — Additional file 1: Supplemental appendix. This appendix provides further detail of model inputs relating to rates of caesarean section delivery, start-up costs for IHO introduction and costs of PPH treatment. [file 12916_2020_1658_MOESM1_ESM.docx]

Cost-effectiveness of inhaled oxytocin for prevention of post-postpartum haemorrhage: a modelling study applied to two high burden settings

Natalie Carvalho, Mohammad Enamul Hoque, Victoria L. Oliver, Abbey Byrne, Michelle Kermode, Pete Lambert, Michelle P. McIntosh, Alison Morgan

Supplemental appendix

This appendix provides further details of model inputs that underlie those listed in the manuscript.

# Appendix table 1: Rates of Caesarean section delivery at each level of care

|  | Bangladesh | | Ethiopia | |
| --- | --- | --- | --- | --- |
|  | Value | Source | Value | Source |
| **Delivery setting 1** Tertiary level public facilities | 69.4% | Health Bulletin (28) | 3.0% | EmONC assessment 2016 (17) |
| **Delivery setting 2**Secondary level public facilities | 23.8% | Health Bulletin (28) | 0.0% | EmONC assessment 2016 (17) |
| **Delivery setting 3** Primary health facilities and non-facility births attended by a skilled provider | 0.0% | Health Bulletin (28) | 0.0% | Assumption |
| **Delivery setting 4** Non-facility births not attended by a skilled provider | 0.0% | Assumption | 0.0% | Assumption |
| **Delivery setting 5** Private facilities | 83.0% | BMMS 2016 (13) | 49.0% | Assumption |

EmONC = Emergency Obstetric and Neonatal Care; BMMS = Bangladesh Maternal Mortality Survey;

# Appendix table 2: List of costs estimates used to calculated total start-up costs for introduction of an inhaled oxytocin product in Bangladesh. All costs are listed in USD (2017)

| **Advocacy** |  |  |  |  |  |
| --- | --- | --- | --- | --- | --- |
| **National level** | **# units** | **unit type** | **USD/unit** | **Total USD** | **Source** |
| Venue hire | 1 | venue | 1,859.89 | 1,859.89 | MoH informant |
| Per diem (participant) | 20 | person | 37.20 | 743.96 | MoH informant |
| Per diem (consultant) | 10 | person | 62.00 | 619.96 | MoH informant |
| Banner | 1 | banner | 123.99 | 123.99 | MoH informant |
| Food cost | 30 | meals | 4.34 | 130.19 | MoH informant |
| **Sub-national level** | **# units** | **unit type** | **USD/unit** | **Total USD** |  |
| Venue hire | 2 | venue | 371.98 | 743.96 | MoH informant |
| Per diem | 56 | person | 18.60 | 1,041.54 | MoH informant |
| Per diem | 2 | person | 31.00 | 62.00 | MoH informant |
| Food cost | 58 | meals | 1.49 | 86.30 | MoH informant |
| **Community level** | **# units** | **unit type** | **USD/unit** | **Total USD** |  |
| Courtyard meetings | 2443 | meetings | 1.24 | 3,029.14 | MoH informant |
| **Training** |  |  |  |  |  |
| **Materials development** | **# units** | **unit type** | **USD/unit** | **Total USD** | **Source** |
| Consultant | 10 | days | 309.98 | 3,099.81 | MoH informant |
| Meeting venue | 1 | venue | 619.96 | 619.96 | MoH informant |
| Per diem | 20 | participants | 37.20 | 743.96 | MoH informant |
| Food | 20 | meals | 1.86 | 37.20 | MoH informant |
| **Master TOT training^a^** | **# units** | **unit type** | **USD/unit** | **Total USD** |  |
| National level | 1 | group session | 12399.26 | 12,399.26 | Health sector plan (16) |
| District level | 64 | group session | 1239.93 | 79,355.24 | Health sector plan (16) |
| **Health worker training** | **# units^d^** | **unit type** | **USD/unit** | **Total USD** |  |
| Up-front training^b^ | 2377 | health facility | 219.85 | 522,591.56 | Health sector plan (16) |
| Refresher training^c^ | 2377 | health facility | 6.43 | 15,282.31 | Community health plan (29) |

MoH = Ministry of Health; TOT = Training of trainers

^a^ Drew on costs of “Capacity Building Orientation for members of DTCC & DUTT” provided in costed health sector plan (page 129)

^b^ Modelled as a half-day on-site group training session at each health facility. Number of health facilities taken from 2016 Health Bulletin. Cost per facility estimated based on costs of “Local training for new vaccine introduction” provided in costed health sector plan (page 46)

^c^ Modelled as a supportive supervision session conducted at each health facility. Drew on costs of “Refresher training of CHCP, by line directors” provided in costed community health sector plan (Annex III(c))

^d^ Number of facilities taken from Health Bulletin, 2016 (1)

# Appendix table 2: List of costs estimates used to calculated total start-up costs for introduction of an inhaled oxytocin product in Ethiopia. All costs are listed in USD (2017)

| Advocacy |  |  |  |  |  |
| --- | --- | --- | --- | --- | --- |
| **National level** | **# units** | **unit type** | **USD/unit** | **Total USD** | **Source** |
| Venue hire | 3 | days | 92.04 | 276.12 | MoH informant |
| Per diem (participant) | 40 | person days | 20.71 | 828.35 | MoH informant |
| Per diem (consultant) | 20 | person days | 20.71 | 414.17 | MoH informant |
| Transport | 15 | persons | 11.50 | 172.57 | MoH informant |
| Banner | 2 | days | 23.01 | 46.02 | MoH informant |
| Food cost | 45 | person days | 6.90 | 310.63 | MoH informant |
| **Sub-national level** | **# units** | **unit type** | **USD/unit** | **Total USD** | **Source** |
| Venue hire | 2 | days | 92.04 | 184.08 | MoH informant |
| Per diem for trainee | 70 | person days | 20.71 | 1,449.61 | MoH informant |
| Per diem for consultant | 6 | person days | 20.71 | 124.25 | MoH informant |
| Transport | 35 | persons | 28.76 | 1,006.67 | MoH informant |
| Consultant | 6 | person days | 115.05 | 690.29 | MoH informant |
| Banner | 2 | days | 23.01 | 46.02 | MoH informant |
| Food cost | 76 | person days | 5.52 | 419.70 | MoH informant |
| **Community level** | **# units** | **unit type** | **USD/unit** | **Total USD** | **Source** |
| Community meetings | 769 | meetings | 46.02 | 35,388.86 | MoH informant |
| **Training** |  |  |  |  |  |
| **Materials development** | **# units** | **unit type** | **USD/unit** | **Total USD** | **Source** |
| Consultant | 60 | person days | 115.05 | 6,902.90 | MoH informant |
| Meeting venue | 10 | days | 92.04 | 920.39 | MoH informant |
| Per diem | 60 | person days | 20.71 | 1,242.52 | MoH informant |
| Food | 80 | person days | 6.90 | 552.23 | MoH informant |
| **Master TOT training** | **# units** | **unit type** | **USD/unit** | **Total USD** | **Source** |
| National level | 1 | group session | 4601.93 | 4,601.93 | MoH informant |
| Region level | 11 | group session | 2300.97 | 25,310.63 | MoH informant |
| **Health worker training** | **# units** | **unit type** | **USD/unit** | **Total USD** | **Source** |
| Up-front training^a^ | 3662 | health facility | 285.32 | 1,044,841.23 | MoH informant, EmONC assessment (17) |
| Refresher training | 3662 | health facility | 39.12 | 143,244.36 | MoH informant, EmONC assessment (17) |

## MoH = Ministry of Health; TOT = Training of trainers; EmONC = Emergency Obstetric and Neonatal Care

## ^a^ Modelled as a half-day on-site group training session at each health facility. Number of health facilities taken from EmONC assessment. Assume HEWs are trained at health centres and not health posts

# Appendix table 3: Unit costs used to estimate cost of PPH treatment in public and private health facilities. All costs are expressed in USD (2017 pricing)

|  | **Public^a^** | | **Private** | |  |
| --- | --- | --- | --- | --- | --- |
|  | **Mild PPH** | **Severe PPH** | **Mild PPH** | **Severe PPH** |  |
| **Bangladesh** |  |  |  |  |  |
| **Medical costs** | **Value** | **Value** | **Value** | **Value** | **Source** |
| Admission fee | 3.54 | 3.54 | 6.20 | 6.20 | Hospital administrators and clinicians |
| Lab test | - | 4.45 | - | 80.61 | Hospital administrators and clinicians |
| Surgical fees^b^ | - | 1.37 | - | 24.8 | Hospital administrators and clinicians |
| Drugs and fluids | 3.54 | 7.08 | 6.20 | 12.4 | Hospital administrators and clinicians |
| Blood transfusion | 8.50 | 17.71 | 14.88 | 31.00 | Hospital administrators and clinicians |
| Bed charge | 17.00 | 42.51 | 29.76 | 74.40 | Hospital administrators and clinicians |
| Service charge | 4.25 | 10.63 | 7.44 | 18.60 | Hospital administrators and clinicians |
| Consultation fee | 9.92 | 24.80 | 17.36 | 43.40 | Hospital administrators and clinicians |
| **Non-Medical costs** | **Value** | **Value** | **Value** | **Source** | **Source** |
| Food | 7.44 | 18.60 | 7.44 | 18.60 | Data previously collected by author (30) |
| Transport | 7.07 | 14.13 | 10.86 | 21.71 | Data previously collected by author (30) |
| Other^c^ | 10.83 | 21.67 | 9.65 | 19.3 | Data previously collected by author (30) |
| **Ethiopia** |  |  |  |  |  |
| **Medical costs** | **Value** | **Value** | **Value** | **Value** | **Source** |
| Admission fee | 0.46 | 0.46 | 3.91 | 3.91 | Akalu et al. 2012 (31) |
| Lab test | 3.05 | 4.55 | 10.65 | 15.90 | Akalu et al. 2012 (31) |
| Surgical fees^b^ | - | 39.00 | - | 457.62 | Pearson et al. 2011 (32) |
| Drugs and fluids | 9.14 | 13.64 | 12.94 | 19.32 | Akalu et al. 2012 (31) |
| Blood transfusion^d^ | - | 135.86 | - | 176.62 | Lara et al. 2007 (33) |
| Bed charge | 11.44 | 28.61 | 14.80 | 36.99 | WHO CHOICE |
| Consultation fee | 1.51 | 2.25 | 3.05 | 4.55 | Akalu et al. 2012 (7) |
| **Non-Medical costs** | **Value** | **Value** | **Value** | **Value** | **Source** |
| Transport | 2.73 | 5.47 | 6.82 | 13.64 | Akalu et al. 2012 (31) |
| Other^c^ | 2.27 | 4.55 | 20.45 | 40.89 | Akalu et al. 2012 (31) |

## PPH = Postpartum haemorrhage

## ^a^ Public sector medical costs in Bangladesh calculated to be 57% of private sector prices based on literature reports of ratio of public to private OOPE (Rahman M, et al. International quarterly of community health education. 2012;33(2):143-57.)

## ^b^ Surgical fees applied to 9.7% and 9.6% of care-seeking cases in Bangladesh and Ethiopia respectively, to reflect that only a proportion of PPH cases result in surgery (assume 5% of all PPH cases result in surgery and only severe cases lead to surgery)

^c^ Represent any other costs incurred when seeking treatment, and includes accommodation for accompanying relatives, tips and other gratuities

## ^d^Assume two units of blood are delivered for severe PPH
